# Stress-related consequences of the coronavirus disease 2019 pandemic on symptoms of Crohn’s disease

**DOI:** 10.1097/MEG.0000000000002081

**Published:** 2021-01-27

**Authors:** Sarah M. Goodday, Simon Travis, Alissa Walsh, Stephen H. Friend

**Affiliations:** a 4YouandMe, Seattle, Washington, USA; b Department of Psychiatry, University of Oxford; c Translational Gastroenterology Unit, NIHR Oxford Biomedical Research Centre, Oxford University Hospitals NHS Foundation Trust, Oxford, UK

**Keywords:** coronavirus disease 2019, stress, Crohn’s disease

## Abstract

**Objectives:**

A link between stress and Crohn’s disease activity suggests an association, but results have been conflicting. The purpose of this study was to assess whether the stress related to the coronavirus disease 2019 (COVID-19) pandemic affected disease activity in patients with Crohn’s disease.

**Basic methods:**

An anonymous survey was distributed to patients through gastroenterology clinics and networks. Patients were asked to report their Crohn’s disease symptoms in the months prior to the COVID-19 pandemic and again during the early stages of the COVID-19 pandemic using the Manitoba inflammatory bowel disease index in addition to questions about stress, perception of reasons for symptom change and personal impact.

**Main results:**

Out of 243 individuals with a confirmed diagnosis of Crohn’s disease, there was a 24% relative increase in active symptoms between the pre-COVID-19 period to the during-COVID-19 period (*P* < 0.0001) reflecting an absolute change from 45 to 56%, respectively. The most frequent reported reason for a change in symptoms was ‘Increased stress/and or feeling overwhelmed’ (118/236), and personal impact of the pandemic was, ‘I’m worrying a lot about the future’ (113/236), both reported by approximately half of respondents.

**Principal conclusions:**

This study serves as a ‘proof of concept’ demonstrating the impact of a significant and uniquely uniform stressor as a natural experiment on Crohn’s disease activity. The severity of symptoms of Crohn’s disease increased during the COVID-19 pandemic. The primary reported reason for symptom change was an increase in stress, not a change in diet, exercise or other lifestyle behaviours, corroborating the hypothesis that stress affects Crohn’s disease activity.

## Introduction

Crohn’s disease has had an increasing global prevalence over the past few decades, with a rising incidence in newly industrialised countries [1, 2]. This increase in incidence may be a result of modernisation, the adoption of westernised diets and lifestyles with consequent changes in the gut microbiota; however, stress is also a plausible explanation. While the aetiology of Crohn’s disease remains unclear, it is posited that the condition arises from complex interactions between genetics, gut microbiota, environment and psychosocial factors [3]. Research on the link between stress and Crohn’s disease activity suggests a correlation [4–7], but findings have been conflicting [8]. The bulk of support for this link stems from indirect evidence of the incidence of psychiatric comorbidities and psychological disturbances in patients with Crohn’s disease, or linking different facets of stress (e.g., early adversity, life events) to disturbances in physiology thought to affect Crohn’s disease, such as neuroendocrine or immune systems [9]. Major depression and Crohn’s disease may share similar biological pathways involving elevated proinflammatory cytokines and oxidative stress [5]. Immune and neuroendocrine dysfunction may be the stress exposure-end organ damage pathway for several diseases [10], but in no condition is this link well understood.

The largest challenge in the stress–disease relationship lies in the measurement of stress, that has varied definitions and approaches to measurement, most of which do not account for the dynamic and individual nature of stress and how individuals react to it [11], or the potential bidirectional relationship between stress and Crohn’s disease. Furthermore, traditional approaches rely heavily on the subjective measurement of stress with long recall periods, or in controlled, unnatural settings. There is a poor understanding of whether perceived stress maps onto objective measures of stress, such as autonomic output, or any physiological damage thought to exacerbate symptoms of Crohn’s disease that might involve immune and neuroendocrine dysfunction, disturbances in intestinal barrier function or microbiota [12].

The novel coronavirus disease 2019 (COVID-19) pandemic has resulted in a substantial strain on individuals, families and communities through major clinical, social and economic constraints. This global phenomenon reflects a unique natural experimental condition where large proportions of the population have faced a surge of complex stressors, such as shifts in a healthy lifestyle, new financial strain and instability, heightened health risks and reduced social connectedness, exposing individuals to excessive stress beyond that already present in their lives. Events of global significance have previously been used in this way, such as world wars [13], or disasters such as Chernobyl [14]. One study examined the stress-related impact of the Great East Japan Earthquake (2011) and interestingly demonstrated an increase in symptoms of ulcerative colitis symptoms, but no change in Crohn’s disease [15].

Global stressful events produce an opportunity to investigate the impact of both common and distinct stressors occurring in the natural environment across different chronic conditions, without having to rely on an individual’s perceptions of stress. Our aim was to use the 2020 pandemic to compare self-reported symptoms of Crohn’s disease pre-COVID-19 and during-COVID-19, to determine whether external stress is associated with changes in disease activity and to understand how the pandemic has affected patients with Crohn’s disease.

## Methods

A short, anonymous survey, was distributed to patients through international gastroenterology clinics and networks via the Leona M. and Harry B. Helmsley Charitable Trust and Oxford University Hospitals (Crohn’s disease patients registered with the Inflammatory Bowel Disease Biobank in Oxford, England). This survey retrospectively assessed symptoms of Crohn’s disease in the months prior to COVID-19 (January and early February 2020) and again during the early stages of the COVID-19 pandemic (March and April 2020). The WHO announced the outbreak of COVID-19 a pandemic on 11 March 2020 and lock-down restrictions started for many countries, continuing into April 2020. We chose these months because they reflect the early, uncertain phase of the pandemic which we speculated might be tied to the most stress. January and ‘early’ February were chosen as the pre-COVID-19 phase because most countries (excluding China and Korea) appeared unaffected by COVID-19, although if they were, it was unknown and not in the media. The survey was live for approximately 8 weeks, between 30 April and 26 June 2020.

### Measures

We used the Manitoba inflammatory bowel disease (IBD) index [16] as a measure of disease activity. The Manitoba IBD index is a single item, 6-level self-reported measure of Crohn’s disease symptoms in the past 6 months. It prompts respondents to select one of the following options: (1) constantly active, giving me symptoms every day; (2) often active, giving me symptoms most days; (3) sometimes active, giving me symptoms on some days (for instance 1–2 days/week); (4) occasionally active, giving me symptoms 1-2 days/month; (4) rarely active, giving me symptoms on a few days; (5) I was well, what I consider a remission or absence of symptoms. The Manitoba IBD index has shown high sensitivity to other validated measures of Crohn’s disease symptom severity, excellent test–retest reliability and strong convergent validity [16], as well as being adopted as the patient-reported measure of disease activity by the International Consortium on Health Outcome Measures (www.ichom.org [17]). For the purposes of this survey, we asked respondents to recall their symptoms during specific periods before and during the COVID-19 pandemic. To calculate percent increases in disease activity, we defined active symptoms as those reported either constantly active, often active, sometimes active or occasionally active and inactive symptoms as rarely active, or well, according to the index recommendations [16]. Bespoke categorical measures were created by asking respondents to report their perceived reason for symptom change, personal impact of the COVID-19 pandemic and self-reported stress levels (very stressed, stressed, neutral, calm, very calm, other).

### Statistical analysis

Descriptive analyses comprising frequencies of symptom severity during the different time periods, stress and COVID-19 specific items were conducted. Changes in symptom severity of ordinal response categories were assessed using nonparametric Wilcoxon sign-rank tests. *Z* scores for Wilcoxon sign-rank tests are not presented owing to the challenges in interpreting these scores with ordinal response data. Changes in active vs. nonactive symptoms were assessed using chi-squared tests or Fisher’s exact tests, where appropriate. All analyses were conducted using IBM SPSS Statistics for Windows, version 26 [18].

## Ethical considerations

This survey was conducted anonymously. Participants were informed of the nature and purpose of the survey in a cover letter and agreed to participate. No personally identifiable information or protected health information was collected from participants from the outset. UK-based participants were part of the UK-Biobank and had consented to be contacted for research and Health Research Authority based decision tools (http://www.hra-decisiontools.org.uk/ethics/) deemed this study exempt from review.

## Results

### Characteristics of the sample

A total of 243 individuals with a diagnosis of Crohn’s disease completed the survey. Almost 100% (242/243) confirmed that their diagnosis was confirmed by a clinical procedure with their doctor (e.g., colonoscopy, under endoscopy, barium or capsule study, computed tomography scan, MRI, or other), while one respondent indicated their diagnosis was self-reported. Most respondents were from the United Kingdom (86%, 209), while 10% (23) were from the United States. Other respondents (<5%) were from Canada, South Africa, New Zealand, Italy or India.

### Impact of the pandemic on Crohn’s disease symptoms

**Fig. 1.**
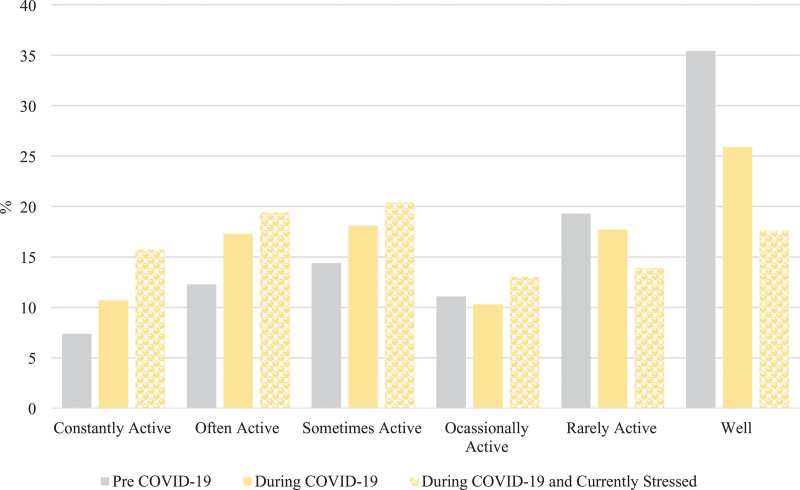
Severity of self-reported Crohn’s disease symptoms pre- and during-COVID-19 (*n* = 243), and in those reporting current stress (*n* = 122). COVID-19, coronavirus disease 2019.

There was a difference in Crohn’s disease symptom severity in the pre-COVID-19 period compared to during-COVID-19 period (Wilcoxon rank-sign test *P* < 0.0001) (Fig. 1). A total of 17% (40/243) of respondents reported a change from inactive to active Crohn’s disease symptoms (Table 1). In particular, there was a 25% relative increase in active Crohn’s disease symptoms between the pre-COVID-19 period compared to the during-COVID-19 period (χ^2^ = 83, *P* < 0.0001), reflecting an absolute change from 45 to 56%. The relative percent increase in active symptoms was more pronounced (42%) among those reporting current stress (*n* = 122) (χ^2^ = 23, *P* < 0.0001) reflecting an absolute change from 48 to 69% from the pre-COVID-19 to the during-COVID-19 periods (Fig. 1).

**Table 1.** Proportion of respondents reporting active vs. inactive Crohn’s disease symptoms pre- and during-COVID-19 (*n* = 243)

	Yes		No	
	*n*	%	No	%
Active^a^ pre-COVID-19	110	45	133	55
Active during-COVID-19	137	56	106	44
Became active	40	17	203	84
Became inactive	13	5	230	95
Stayed active	97	40	146	60
Stayed inactive^a^	93	38	150	62

a According to Manitoba IBD index: active = constantly active, often active, sometimes active or occasionally active; inactive = rarely active or well.

COVID-19, coronavirus disease 2019; IBD, inflammatory bowel disease.

### Reasons for symptom change in Crohn’s disease during the COVID-19 pandemic

**Fig. 2.**
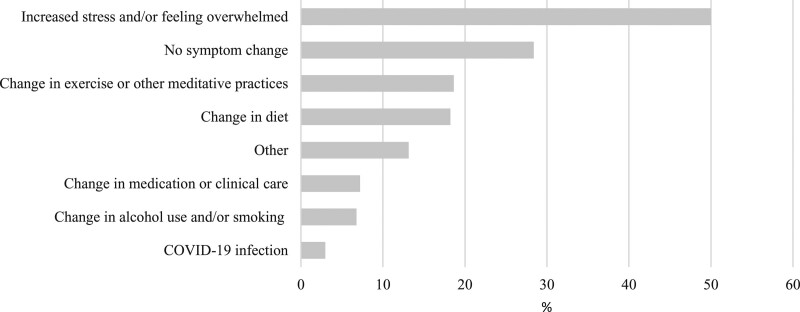
Reported reasons for symptom changes in Crohn’s disease (*n* = 236). COVID-19, coronavirus disease 2019.

**Fig. 3.**
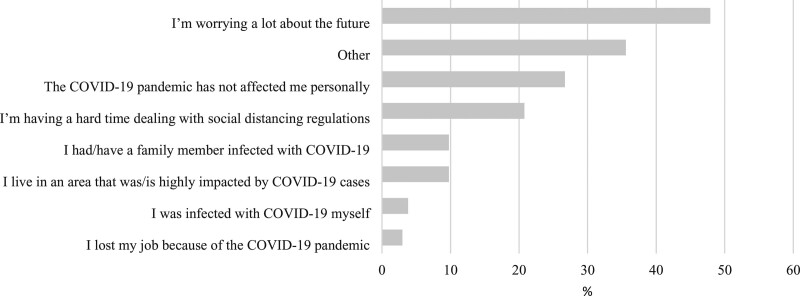
Reported personal impact of the COVID-19 pandemic (*n* = 236). COVID-19, coronavirus disease 2019.

The most frequent reported reason for a change in symptoms from the pre- to during-COVID-19 periods was ‘Increased stress/and or feeling overwhelmed’ (50%, 118/236) (Fig. 2). The most frequently reported personal impact of the pandemic was ‘I’m worrying a lot about the future’ (48%, 113/236) (Fig. 3). A total of 36% (84/236) of respondents indicated ‘other’ reasons in free text. The majority of ‘other’ reasons were comparable to the existing categories and largely comprised the following: suspected COVID-19, worry about family and friends, social isolation, stress from homeschooling children and work/finance-related issues.

**Fig. 4.**
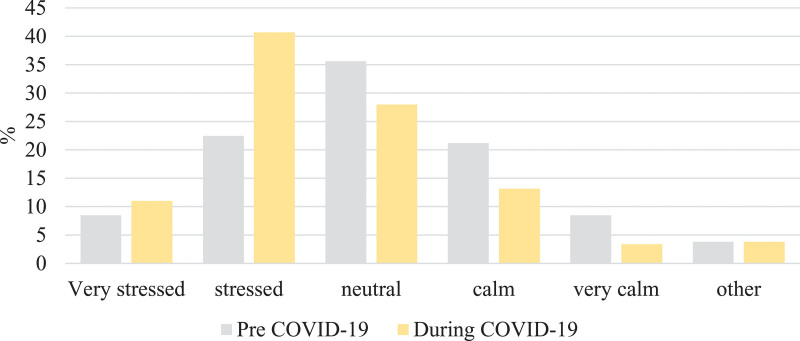
Proportion of respondents reporting stress before and during the COVID-19 pandemic. COVID-19, coronavirus disease 2019.

Respondents were asked to report their stress levels during the two time periods, to corroborate the increase in perceived stress. The proportion of respondents feeling ‘stressed’ of ‘very stressed’ increased from 31% (73/236) in the pre-COVID-19 period to 52% (122/236) in the during-COVID-19 period, a relative increase of 40% (Fig. 4).

**Fig. 5.**
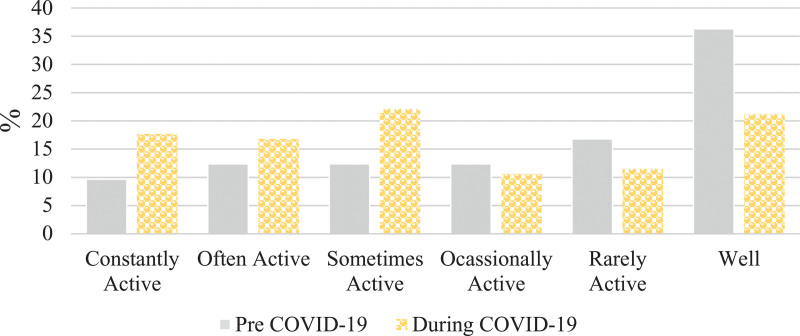
Severity of self-reported Crohn’s disease symptoms pre- and during-COVID-19 in those reporting worry about the future (*n* = 113/243). COVID-19, coronavirus disease 2019.

In a subset of respondents (113/243) reporting increased worry about the future, the severity of Crohn’s disease symptoms changed between the pre- and during-COVID-19 periods (Fig. 5) (Wilcoxon signed-rank test, *P* < 0.0001). The relative increase in active symptoms of Crohn’s disease was more pronounced in this subset reporting worry (43%, chi-square = 22, *P* < 0.0001) reflecting an absolute change from 47 to 67% from the pre-COVID-19 to the during-COVID-19 period.

## Discussion

The severity of symptoms of Crohn’s disease significantly increased during the COVID-19 pandemic, and the primary perceived reason for symptom change was an increase in stress. Respondents reported feeling overwhelmed as the primary reason, not any change in diet, exercise or other lifestyle behaviours. This corroborates the hypothesis that stress affects Crohn’s disease activity. During the early, uncertain months of the pandemic (March–April 2020), over half of the 243 respondents reported symptoms of active Crohn’s disease while in those reporting worry about the future and current stress, over two-thirds reported active symptoms. This represents a 25% increase in symptoms of active Crohn’s disease in the whole sample and a more than 40% increase in those reporting current stress or worries about the future.

Studies linking stress to Crohn’s disease have been conflicting, which is in part due to the challenge of measuring stress in the natural environment. The COVID-19 pandemic provides a unique opportunity to conduct a natural experiment where almost all individuals are facing both common and distinct stressors relating to social constraints, isolation, shifts in lifestyle, access to medical care, or major financial challenges. An important stressor to recognise is the unpredictability of the pandemic, with varied and disjointed government responses, unknowns about current or future lock-down restrictions, the effect of an underlying disease or its treatment on COVID-19 and about the future itself. The largest reported impact from the COVID-19 pandemic in our survey was ‘increased worry about the future’. We recognise the ambiguity in this question, which encompasses both worries about short-and long-term events. However, we were interested in the general concept of worry, irrespective of nature, as the pandemic has affected individuals very broadly, in a way that is difficult to capture in a single or even across several items. Theories of stress and disease are many, but recent theories [19] suggest that perceptions of uncertainty, unpredictability or lack of control may be at the root of what stress constitutes and the driving external source of the consequences of stress. The nature of stress faced by many people as a result of the COVID-19 pandemic is in line with uncertainty as the principal source of stress, which is disconcerting not only for people with Crohn’s disease, but for those suffering from many chronic conditions affected by stress.

This study has a number of limitations. It is retrospective and based on self-reported survey data. Recall and other information and selection biases cannot be excluded. However, we used a symptom measure of disease activity (the Manitoba IBD index) with strong psychometric properties that has a validated recall over the preceding 6 months, and we did not ask respondents to recall symptoms beyond this time frame. Further, the survey was not framed as a study on stress; therefore, we do not expect that responding participants would have had a priori assumptions about our hypothesis. We assumed that respondents experienced a change in stress primarily as a consequence of the COVID-19 pandemic during the specified time frames. Given the unprecedented nature of the COVID-19 pandemic, a natural increase in stress in most individuals was a reasonable assumption and respondents reported a significant increase in self-reported stress during the COVID-19 period. Most of our respondents were from the UK. We expect this was a function of the higher distribution to UK-based patient lists that comprised patients already registered and engaged in research (e.g., the IBD Biobank) involving digital technology.

Physiological factors have rarely been studied alongside objective measures of stress in Crohn’s disease, in part due to methodological challenges of measuring these domains in real-time. With the ubiquity of digital devices, including smartphone active and passive sensing, wearable devices, such as smartwatches, rings or body tags and patches, an opportunity exists to better understand the links between individual stress levels and Crohn’s disease activity [11]. Multiple digital devices are able to track multimodal measures of stress, including semicontinuous autonomic output such as heart rate, heart rate variability and respiratory rate, and possibly objective, intermediate signs of stress such as sleep, mood and cognition. More importantly, these devices can capture the individual context giving rise to these symptoms [11]. A high-resolution digital approach, coupled with machine learning and artificial intelligence provides a potential solution to inform the complex relationships between stress, as it occurs in real-time and Crohn’s disease activity.

### Conclusion

Stress related to the COVID-19 pandemic significantly increased symptoms of active Crohn’s disease by 25%. This study serves as a ‘proof of concept’ to demonstrate the impact of a significant stressor as a natural experiment on Crohn’s disease activity. Our findings show that a significant proportion (more than 40%) of respondents reported symptoms of active Crohn’s disease during the beginning of the COVID-19 pandemic, showing a significant increase in severity of symptoms from earlier months, prior to the pandemic. Methodological challenges in detecting and tracking stress, while accounting for individual differences will continue to create a barrier in the stress–Crohn’s disease research field. A multimodal digital approach, taking advantage of advances in wearable devices and smartphone sensors is likely to gain insight into the way stress might exacerbate Crohn’s disease.

## Acknowledgements

We would like to thank the Leona M. and Harry B. Helmsley Charitable Trust and Oxford University Hospitals for distributing this survey to Crohn’s disease networks and Gastroenterology clinics.

## Conflicts of interest

There are no conflicts of interest.

This work was supported by 4YouandMe and in part by the National Institute for Health Research (NIHR) Oxford Biomedical Research Centre (BRC). The views expressed are those of the author(s) and not necessarily those of the NHS, the NIHR or the Department of Health.
